# The Role of CHPD and AIMI processing on enhancing *J*_C_ and transverse connectivity of *in-situ* MgB_2_ strand

**DOI:** 10.1088/1757-899x/756/1/012018

**Published:** 2020-06-29

**Authors:** F Wan, M D Sumption, M A Rindfleisch, E W Collings

**Affiliations:** 1 Center for Superconductor and Magnetic Materials, Department of Materials Science and Engineering, The Ohio State University, Columbus, OH 43210, USA.; 2 Hyper Tech Research Inc, Columbus, OH 43228, USA

## Abstract

Research into *in-situ* MgB_2_ strand has been focused on improvements in *J*_C_ through reduction of porosity. Both of cold-high-pressure-densification (CHPD) and advanced-internal-magnesium-infiltration (AIMI) techniques can effectively remove the voids in *in-situ* MgB_2_ strands. This study shows the nature of the reduced porosity for *in-situ* MgB_2_ strands lies on increases in transverse grain connectivity as well as longitudinal connectivity. The CHPD method bi-axially applying 1.0 GPa and 1.5 GPa yielded 4.2 K *J*_CM∥_s of 9.6 × 10^4^ A/cm^2^ and 8.5 × 10^4^ A/cm^2^ at 5 T, respectively, with compared with 6.0 × 10^4^ A/cm^2^ for typical powder-in-tube (PIT) *in-situ* strand. Moreover, AIMI-processed monofilamentary MgB_2_ strand obtained even higher *J*_C_s and transverse grain connectivity than the CHPD strands.

## 1. Introduction

MgB_2_ superconducting strands are promising to the practical magnetic application due to its high transition temperature *T*_*C*_ (39 K) [1], high coherence length [2, 3], and low anisotropy of upper critical fields (*B*_*c2*_) [3–6]. The powder-in-tube (PIT) *in-situ* MgB_2_ strands were fabricated by filling a mixture of Mg and B powder into a non-reactive metallic tube and then being cold-worked into wires or tapes. The PIT strands have large amount of voids elongated along longitudinal strand axis which were left behind by molten Mg powders after heat treatment [7]. The present of voids tends to limit the number of the continuous current path in the *in-situ* MgB_2_ strand and therefore suppress the current-carrying-capacity of the strand.

The CHPD technique can effectively increase the transport properties of PIT *in-situ* MgB_2_ strands by eliminating the pores [8–11]. The pre-reacted powder-in-tube composite was bi-axially cold-densified at room temperature to increase the mass density of the Mg + B mixture. In this case, the cold-densified MgB_2_ strand can obtain higher grain connectivity after heat treatment. Additionally, the Mg reactive – liquid – infiltration (RLI) process, initiated by Giunchi *et al* [12], also has the ability to eliminate the pores and produce a dense MgB_2_ layers in *in-situ* MgB_2_ strands. For RLI process, a Mg rod is inserted axially into a boron-filled metallic tube. After wire drawing the heat treatment (H. T.), MgB_2_ layer is formed through the reactive diffusion of Mg into B layer. Since Mg is totally separated with precursor B layer before H. T., the RLI process can totally eliminate the “Mg-site porosity” from MgB_2_ layer but induce the formation of a big hole at the central region of the strand [13]. Furthermore, since the molar volume of MgB_2_ (17.46 cm^3^/mol) is twice as that of two B atoms (9.18 cm^3^/mol), the volume expansion associated with the reactive transformation from 2B to MgB_2_ enables even better connections between MgB_2_ grains during heat treatment [13]. Our group named our RLI-processed strands as advanced – internal – magnesium – infiltration (AIMI) strands due to their optimized strand architecture and high *J*_C_s [13].

The previous researches mostly focused on investigating the effect of CHPD and AIMI techniques on the transport properties along longitudinal strand axis of *in-situ* MgB_2_ strands, such as transport *J*_C_s and longitudinal grain connectivity. However, Shi and Susner pointed out that high anisotropic grain connectivity exists in PIT *in-situ* strands, which was resulted from the elongated voids between elongated MgB_2_ stringers [7]. Therefore longitudinal transport properties are different with the transport properties along transverse strand axis for MgB_2_ superconducting wires. In continuing along these lines, the transport properties along transverse strand axis were investigated for the 2.0 mol% C-doped *in-situ* MgB_2_ strands in this study. The anisotropic connectivity of the PIT strands results in the differences between perpendicular magnetic *J*_C_ (*J*_CM_|__) and parallel *J*_C_ (*J*_CM∥_). The influence of aspect ratio (S = length/diameter) on transverse and longitudinal *J*_C_ were investigated for the PIT strand (P00). The CHPD-processed strands were P10 (1.0 GPa cold-pressing) and P15 (1.5 GPa cold-pressing). According to the previous results of our group, the CHPD technique increased the transport *J*_C_ of the monofilamentary PIT *in-situ* MgB_2_ strand from 3.0 × 10^4^ A/cm^2^ to 3.6 × 10^4^ A/cm^2^ at 4.2 K and 10 T due to decreased porosity [11] and AIMI-processed MgB_2_ strands attained the 4.2 K, 10 T transport layer *J*_C_s of 1.0 ~ 1.5 × 10^5^ A/cm^2^ [13–16]. In this study, we compared the *J*_CM∥_s and transverse connectivity of the CHPD- and AIMI-processed strands with those of the P00 strand at 4.2 K and 20 K. The relationship between porosity and transverse flux pinning force density *F*_p∥_, which is *J*_CM∥_ × *B*, for the *in-situ* MgB_2_ strands was also discussed.

## 2. Experimental

### 2.1. Sample preparation

A series of pre-reacted powder-in-tube (PIT) *in-situ* strands, typically 0.834 mm diameter, with a Nb barrier and a Cu outer sheath were fabricated by Hyper Tech Research, Inc. (HTR). Two PIT strands (designated P10 and P15) were bi-axially densified with 1.0 GPa and 1.5 GPa at room temperature, respectively. The other strand (designed A00) manufactured through AIMI technique were also provided by HTR. The AIMI-processed strand, with 0.55 mm diameter, has a Nb barrier and a Monel outer sheath. The powders used for the present strands were 2 mol% C–doped amorphous B (10 – 100 nm) from Specialty Materials Inc. (SMI). The cold–densified PIT strands were heat-treated at 675 °C for 1 h and the AIMI strand was heat – treated at 625 °C for 16 h. The specification and heat treatment (H. T.) conditions of the strands are presented in table 1.

### 2.2. Transport and Magnetic Measurements

The transport *I*_C_ (*I*_CT_) test was conducted in perpendicular magnetic field up to 13 T in a pool of liquid Helium at 4.2 K on the MgB_2_ strands with a total length of 50 mm and a gauge length of 5 mm. The electric criterion used for determining *I*_CT_s is 1.0 μV/cm. The magnetizations versus perpendicular and parallel magnetic fields (*M* – *H*) loops were measured by a Quantum Design Model 6000 Physical Property Measuring System (PPMS) for all strands with a sample length of 3 – 5 mm.

## 3. Results

**Figure 1.**
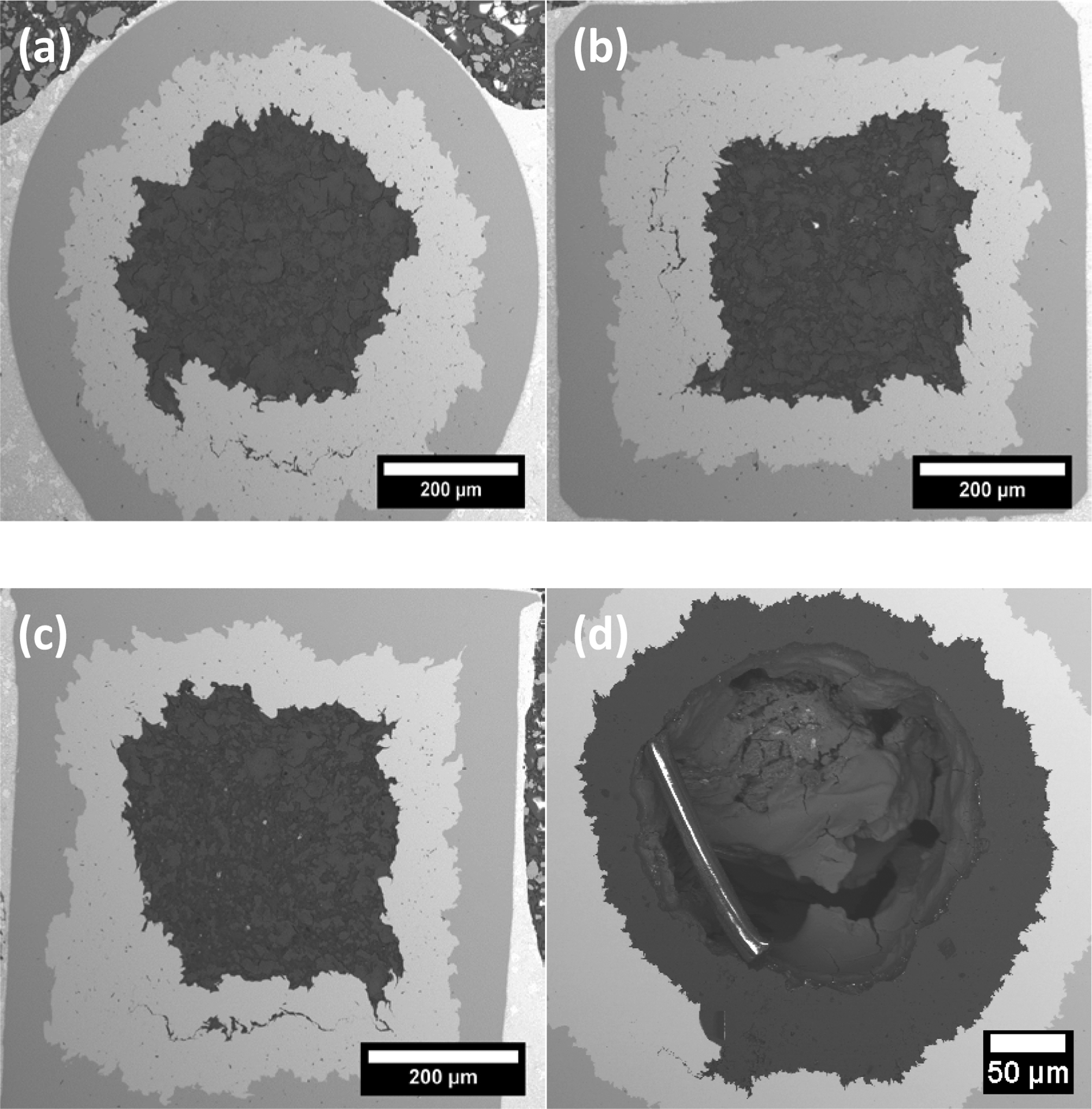
(a) Back scattered SEM images of (a) strand P00, (b) strand P10 (1.0 GPa cold pressure), (c) strand P15 (1.5 GPa cold pressure), and (d) AIMI strand A00.

The *J*_CT_s of the PIT strands were the transport critical current normalized by MgB_2_ core area. As shown in Figure 1(a) the MgB_2_ core of the typical PIT *in-situ* strand is a solid cylinder. Figure1(b)–(d) shows the shape of MgB_2_ cores for the CHPD- and AIMI-processed strands are cuboid and hollow cylinder, respectively. The *J*_CT_ of the AIMI–processed strands, which is also named as transport layer *J*_C_, were calculated by dividing the *I*_CT_ by the area of annulus MgB_2_ layer. Values of magnetic *J*_*C*_ (*J*_CM_|__ and *J*_CM∥_) for the MgB_2_ strands were extracted from the full *M* – *H* loops heights Δ*M*, using the standard Bean model equations [7, 17]:

For the PIT *in–situ* wire P00:
  (1) $$\text{ Perpendicular Magnetic }J_{\text{C}}:\;J_{\text{CM\_|\_}}=\frac{3\pi \Delta M}{8R_{0}}$$
  (2) $$\text{ Parallel Magnetic }J_{\text{C}}:\;J_{\text{CM}\|}=\frac{3\Delta M}{2R_{0}}$$
  Here R_0_ is the radius of the cylinder MgB_2_ core in the PIT wire.
For the densified wires P10 and P15:
  (3) $$\text{ Parallel Magnetic }J_{\text{C}}:\;J_{\text{CM}\|}=\frac{2\Delta M}{b\left(1-\frac{b}{3a}\right)}$$
  Here a, b are both lengths of the transverse cross – sectional area of cuboid MgB_2_ core, a > b.
For the AIMI wire A00:
  (4) $$\text{ Parallel Magnetic }J_{\text{C}}:\;J_{\text{CM}\|}=\frac{3\Delta M}{2}\frac{R_{0}^{2}-R_{i}^{2}}{R_{0}^{3}-R_{i}^{3}}$$
  Here *R*_i_ is the inner diameter of the annulus MgB_2_ layer and *R*_0_ is the outer diameter of the annulus MgB_2_ layer.

### 3.1. Transport and magnetic critical current densities, J_CT_ and J_CM_

**Figure 2.**
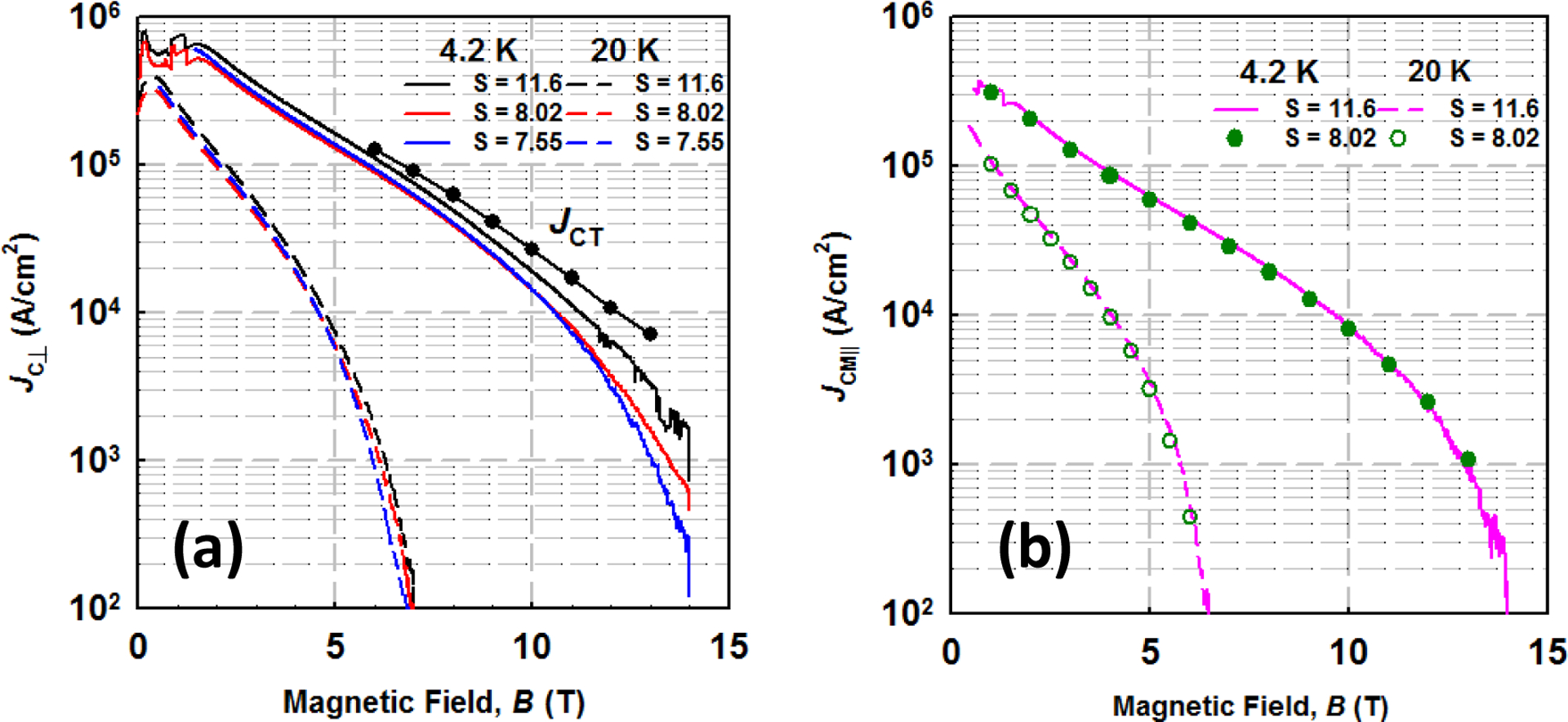
(a) *J*_CT_ and *J*_CM_|__ versus *B* at 4.2 and 20 K (b) *J*_CM∥_ versus *B* at 4.2 and 20 K for strand P00

Figure 2(a) shows the *J*_CT_ and *J*_CM_|__ versus *B* at 4.2 and 20 K for the strand P00. It can be seen that *J*_CM_|__s agree with *J*_CT_ at low fields, whereas the bifurcation of *J*_CT_ and *J*_CM_|__ happened at high fields. Moreover, *J*_CM_|__s were greatly affected by the aspect ratio S, especially at high magnetic fields. The relationships among *J*_CT_, *J*_CM_|__, and aspect ratio were fully discussed in ref [7] and [18]. Therefore, the values of *J*_CM_|__ are not only determined by the intrinsic properties but also the extrinsic properties of the *in-situ* MgB_2_ strands. As shown in Figure 2(b) *J*_CM∥_s were independent on the aspect ratio S. Based on this result, we can know the values of *J*_CM∥_s as well as *J*_CT_s are merely determined by the intrinsic properties of the *in-situ* MgB_2_ strands. The MgB_2_ macrostructure of the reacted PIT *in-situ* strand is characterized by elongated polycrystalline MgB_2_ fibers and elongated pores partially separating the fibers [7, 11, and 18]. The *J*_CT_s and *J*_CM∥_s can represent the longitudinal and transverse connectivity of the MgB_2_ fibers, respectively. In summary, we can investigate the effect of CHPD and AIMI technique on the current–carrying–capacity of the *in–situ* MgB_2_ strands through *J*_CM∥_s as well as *J*_CT_.

**Figure 3.**
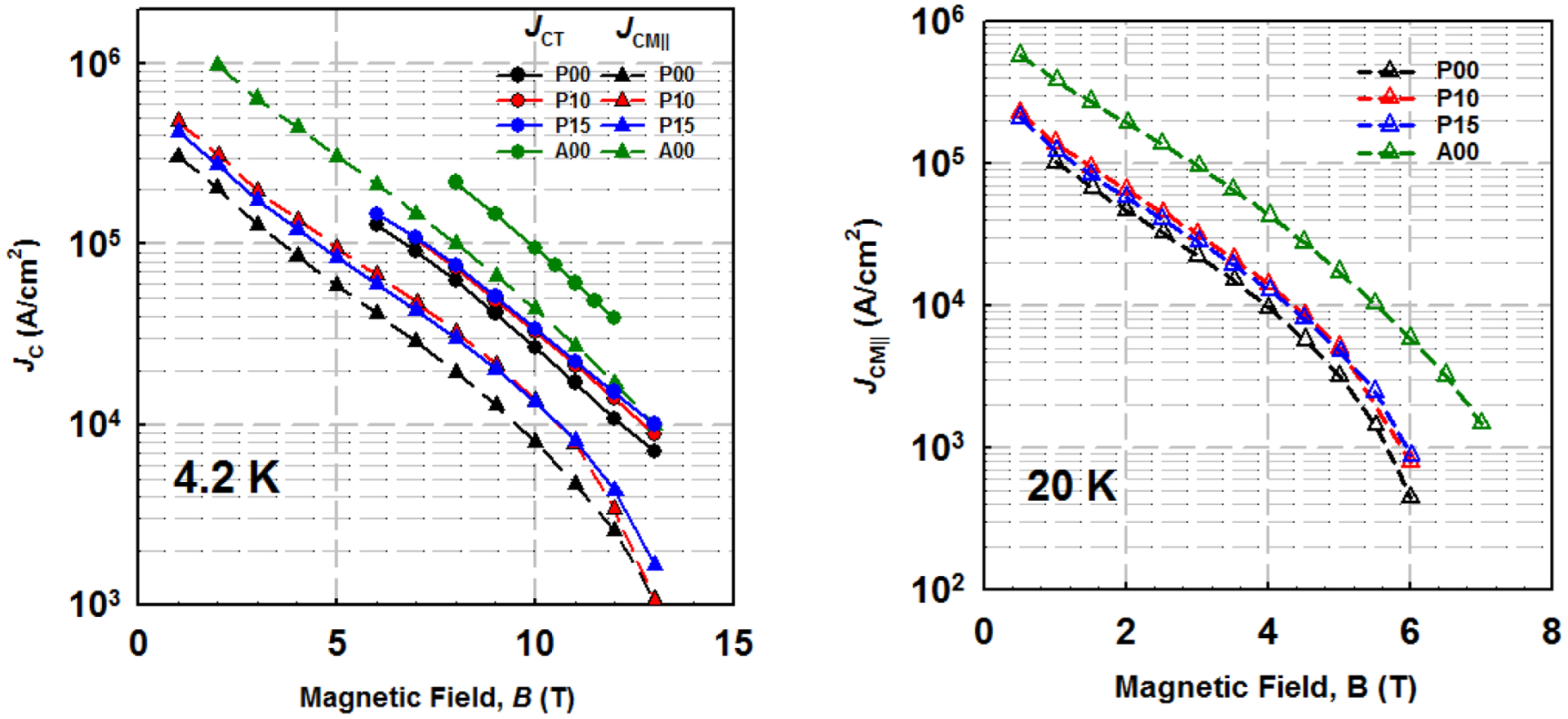
*J*_CT_ and *J*_CM∥_ versus *B* at 4.2 and 20 K for all strands

Figure 3 shows the *J*_CT_ and *J*_CM∥_ versus *B* at 4.2 and 20 K for all strands. The CHPD technique significantly enhanced *J*_CM∥_s of PIT *in-situ* strands at 4.2 K. Strand P10 and P15 attained 9.5 × 10^4^ A/cm^2^ and 8.5 × 10^4^ A/cm^2^ at 4.2 K and 5 T, with respect to 6.0 × 10^4^ A/cm^2^ for strand P00. 4.2 K *J*_CT_s were slightly enhanced by the CHPD technique. Therefore, the increases in *J*_CT_ and *J*_CM∥_ indicated that the both longitudinal and transverse connections between MgB_2_ fibers were enhanced. With the formation of high density MgB_2_ layer, strand A00 attained 4.2 K, 10 T *J*_CT_ of 9.4 × 10^4^ A/cm^2^, which is 180% higher than those of CHPD-processed strands. On the other hand, the AIMI strand obtained 5 T *J*_CM∥_s of 3.1 × 10^5^ A/cm^2^ at 4.2 K and 1.7 × 10^4^ A/cm^2^ at 20 K, which are about 240% higher than those of CHPD strands.

### 3.2. Transverse Flux Pinning, Porosity and Transverse Grain Connectivity

**Figure 4.**
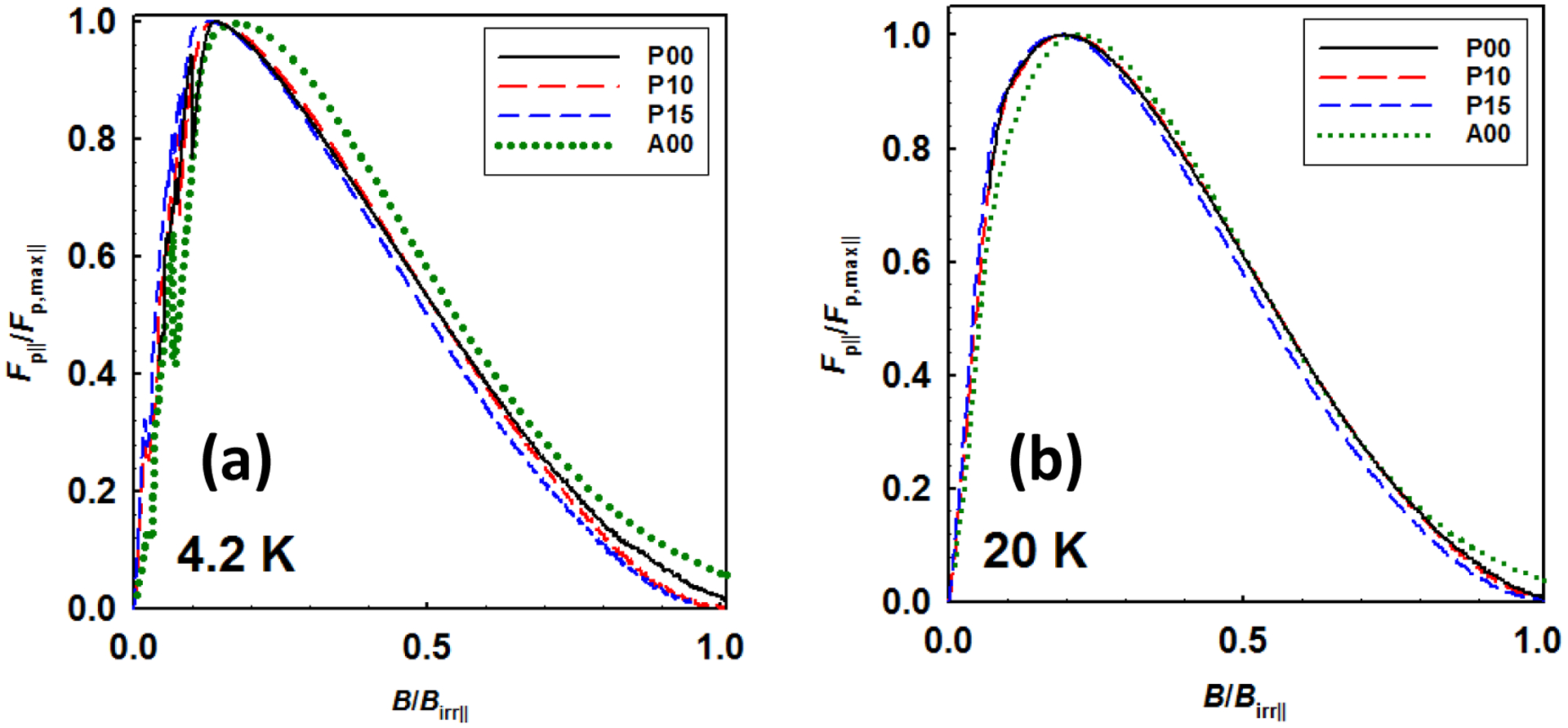
(a) *F*_*p∥*_*/F*_*p,max∥*_ versus *B/B*_*irr*_ for all strands at 4.2 K, (b) *F*_*p∥*_*/F*_*p,max∥*_ versus *B/B*_*irr*_ for all strands at 20K

Figure 4 shows the *F*_p∥_/*F*_p,max∥_ versus *B*/*B*_irr_ for all strands, where *F*_p∥_ = *J*_CM∥_ × *B*. It can be seen that the peak pinning occurred at *b* = *B*/*B*_irr∥_ close to 0.2 at 4.2 and 20 K, which is in agreement with the Dew-Hughes/Kramer model [19, 20]. In other words, the dominant pinning centers for the all strands are also grain boundaries for the direction along transverse strand axis. According to the previous work [11], the densified wires have decreased porosities, the values of porosity are ~ 50% (p = 0) and ~ 30 % (p = 1.0 or 1.5 GPa). Since the Mg–site porosity was totally eliminated for AIMI–processed strands, the porosity of the AIMI strand is close to 0. Therefore, both CHPD and AIMI processes enable the resulting MgB_2_ phases to be denser and more connected. For the discussion, it can be concluded that the enhanced *J*_CT_ and *J*_CM∥_ in densified wires and AIMI wire is correlated with lower porosity (higher grain connectivity).

The connectivity *K* defined by Rowell [21] can represent the grain connectivity of MgB_2_ strands. The connectivity *K* is calculated by the equation:

(5) $$K=\frac{\Delta \rho_{SC}}{\Delta \rho }$$

Here Δ*ρ* is the difference between the sample’s resistivity at 300 K and the sample’s resistivity at 40 K and Δ*ρ*_*SC*_ is the resistivity difference for an ideal single crystal. However, transverse connectivity *K*_∥_ is difficult to be determined for the MgB_2_ wires. It has been reported that the maximum flux pinning force densities of fully–connected MgB_2_ superconductor, where grain connectivity *K* = 100 %, are estimated to be 90 GN/m^3^ at 4.2 K and 22 GN/m^3^ at 20 K [22]. Therefore, we can roughly estimate the transverse grain connectivities *K*_∥_s of the *in-situ* MgB_2_ strands by normalizing the *F*_p,max∥_ with the *F*_p,max_ of fully-connected MgB_2_. Table 2 shows the transverse maximum flux pinning force densities *F*_p,max∥_ at 4.2 K and 20 K and estimated transverse grain connectivities for all the strands. The connectivity of 5% is achieved by the 2 mol% C-doped PIT strand, P00. The cold–densification increases the connectivity of PIT strand by 20 %. The AIMI strand A00 obtained the highest transverse grain connectivities of 20 %.

## 4. Conclusion

*J*_CM∥_s is merely determined by the intrinsic properties of *in-situ* MgB_2_ strands, so the current-carrying capacity of *in-situ* MgB_2_ strands can be represented by *J*_CM∥_ as well as *J*_CT_. The CHPD of 1.0 GPa and 1.5 GPa enhanced the 4.2 K, 5 T *J*_CM∥_ from 6.0 × 10^4^ A/cm^2^ to 9.6 × 10^4^ A/cm^2^ and 8.5 × 10^4^ A/cm^2^, respectively. AIMI strand attained the highest *J*_CT_ and *J*_CM∥_ at 4.2 and 20 K due to the formation of a high dense MgB_2_ layer. By eliminating the voids in *in-situ* MgB_2_ strands through CHPD and AIMI technique, better connections between MgB_2_ grains along transverse strand axis can be obtained in *in–situ* MgB_2_ strands

## Figures and Tables

**Figure 5.**
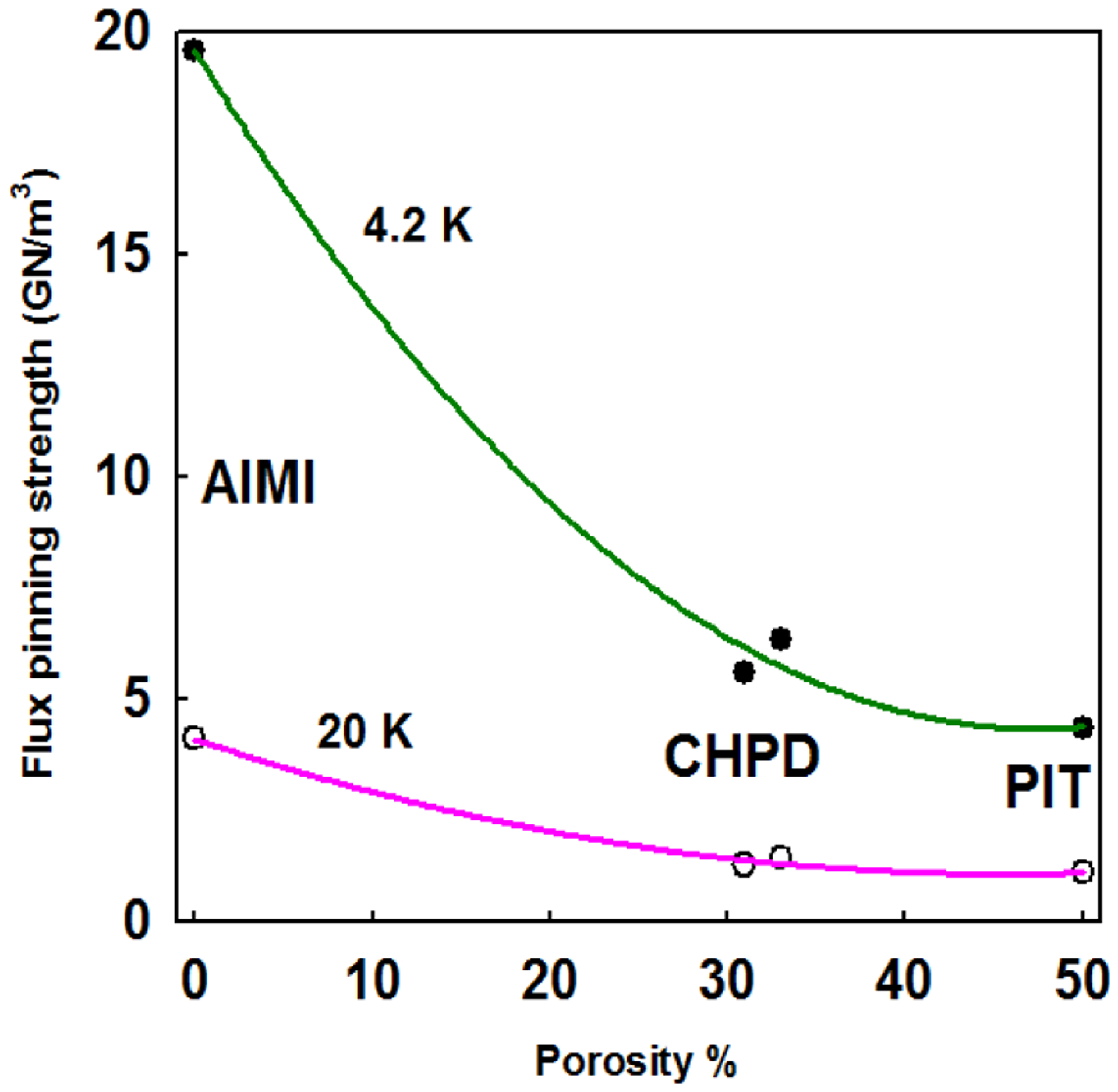
Relationship between porosity and transverse flux pinning force densities for *in–situ* MgB_2_ strands

**Table 1.** Strand specification and H. T. conditions

Strand Name	Strand Type	CHPD (GPa)	H. T. (h/°C)
**P00**	PIT	0.0	1/675
**P10**	PIT	1.0	1/675
**P15**	PIT	1.5	1/675
**A00**	AIMI	0.0	16/625

**Table 2.** Transverse flux pinning force densities and estimated connectivity of the strands.

Strand Name	*F*_p,max∥,_ 4.2 K (GN/m^3^)	*F*_p,max∥,_ 20 K (GN/m^3^)	Transverse Connectivity, *K*_∥_ *%*
**P00**	4.3	1.1	5.0
**P10**	6.3	1.4	6.0
**P15**	5.6	1.3	6.0
**A00**	19.6	4.1	20
